# Regulation of Fig (*Ficus carica* L.) Fruit Color: Metabolomic and Transcriptomic Analyses of the Flavonoid Biosynthetic Pathway

**DOI:** 10.3389/fpls.2017.01990

**Published:** 2017-11-20

**Authors:** Ziran Wang, Yuanyuan Cui, Alexander Vainstein, Shangwu Chen, Huiqin Ma

**Affiliations:** ^1^Department of Fruit Tree Sciences, College of Horticulture, China Agricultural University, Beijing, China; ^2^The Robert H. Smith Faculty of Agriculture, Food and Environment, Institute of Plant Sciences and Genetics in Agriculture, The Hebrew University of Jerusalem, Rehovot, Israel; ^3^College of Food Science and Nutrition Engineering, China Agricultural University, Beijing, China

**Keywords:** fig (*Ficus carica* L.), anthocyanin, flavonoid, peel color mutation, transcriptome, metabolome

## Abstract

Combined metabolomic and transcriptomic analyses were carried out with fig cultivar Green Peel and its color mutant “Purple Peel.” Five and twenty-two metabolites were identified as having significantly different contents between fruit peels of the two cultivars at young and mature stages, respectively. Cyanidin O-malonylhexoside demonstrated a 3,992-fold increase in the mature purple peel, the first identification of a major cyanidin in fig fruit; cyanidin 3-O-glucoside, cyanidin O-malonylhexoside O-hexoside and cyanidin-3,5-O-diglucoside were upregulated 100-fold, revealing the anthocyanins underlying the purple mutation. Beyond the visible differences, there was very significant accumulation of the colorless flavonoids procyanidin B1, luteolin-3′,7-di-O-glucoside, epicatechin and quercetin-3-O-rhamnoside in the mature “Purple Peel” compared to “Green Peel.” At the young stage, only cyanidin O-malonylhexoside, cyanidin O-malonylhexoside O-hexoside and esculetin were upregulated a few fold in the mutant. Transcriptome analysis revealed a downregulated expression trend of genes encoding phenylpropanoid and flavonoid biosynthetic pathway enzyme in the young “Purple Peel” compared to the young “Green Peel,” whereas significant and simultaneous upregulation was revealed in almost all of the flavonoid and anthocyanin pathway components and relevant transcription factors in the mature-stage mutant. The role of R2R3-MYB transcription factors in the color morph mutation and its possible relation to the activity of retrotransposons are discussed. Moreover, large-scale upregulation of small heat-shock protein genes was found in the mature mutant. This is the first work to reveal comprehensive metabolome and transcriptome network changes underlying a fig mutation in a single horticultural attribute, and its profound effects on fruit nutrition and quality.

## Introduction

The fruit peel is in essence the fruit boundary; it maintains fruit integrity and protects it from the external environment. Secondary metabolites in the peel, such as pigments, tannins and aroma compounds, affect fruit appearance, quality and storage (Li et al., 2013). Anthocyanin pigments—pelargonidin, cyanidin, delphinidin, peonidin, petudinin and malvidin, often in their glycosylated form—are commonly identified in pink, red, purple and other deep-colored fruit (Jaakola, 2013).

Anthocyanin metabolism is catalyzed by a number of enzymes from the phenylpropanoid and flavonoid biosynthetic pathways (Bilyk and Sapers, 1986; Pelletier et al., 1997; Falcone Ferreyra et al., 2012). As an initial precursor of anthocyanins and other flavonoids, phenylalanine produces colorless secondary intermediate metabolites that are sequentially catalyzed by phenylalanine ammonia-lyase (PAL), cinnamic acid 4-hydroxylase, 4-coumarate:coenzyme A ligase (4CL), chalcone synthase (CHS), chalcone isomerase (CHI), flavanone 3-hydroxylase (F3H), flavanone 3′-hydroxylase (F3′H), flavonoid 3′5′-hydroxylase, and dihydroflavonol 4-reductase (DFR); unstable colored anthocyanins are then synthesized from the colorless anthocyanins by anthocyanidin synthase (ANS) (Boss et al., 1996; Falcone Ferreyra et al., 2012) via the full metabolic pathway. Finally, the unstable colored anthocyanins are transformed into blue–violet, brick-red or magenta glycosides by UDP-glucose: flavonoid 3-O-glucosyltransferase (UFGT) (Pelletier et al., 1997; Dick et al., 2011; Saito et al., 2013), resulting in different types and numbers of substituents in the B ring of the anthocyanin, which determine the color hue and chromaticity of the anthocyanidins in specific tissues and cellular environments (Espley et al., 2007). Brightly colored fruit commonly show high gene expression of the key downstream enzymes of the anthocyanin biosynthetic pathway, such as those encoding DFR, ANS, and UFGT (Han et al., 2012). Sharply upregulated *FcANS1* expression was revealed in the peel of a dark-colored fig during fruit ripening (Cao et al., 2016), whereas *UFGT* was identified as the critical gene for anthocyanin biosynthesis in grape and strawberry (Kobayashi et al., 2001; Griesser et al., 2008). In recent years, combined high-throughput methods have been used to study color development. Integrated metabolomic and transcriptomic network analyses in fruit and flowers have elucidated a series of secondary metabolites with changes in content, and the corresponding differentially expressed genes (Lou et al., 2014; Matus, 2016), broadening the global view of plant color regulation.

Color mutations are frequently observed in flowers and fruit. The color change is usually produced by single-gene mutations, as in grape (Kobayashi et al., 2004; Hichri et al., 2011a), apple (Xie et al., 2012), pear (Li et al., 2013), and blood orange (Rodrigo et al., 2003). Studies on color mutations have revealed that in addition to the aforementioned structural biosynthetic genes, transcription factors play important roles in modulating anthocyanin biosynthetic pathway activity and color changes (Lepiniec et al., 2006; Saito et al., 2013). The MBW complex [MYB transcription factor in a complex with basic helix-loop-helix (bHLH) and WD40 proteins] has been shown to regulate the expression of anthocyanin genes (Ramsay and Glover, 2005; Petroni and Tonelli, 2011). In the model plant *Arabidopsis*, MYB transcription factors TT2, PAP1/PAP2, MYB75, MYB90, MYB113, and MYB114, bHLH transcription factors TT8, GL3, and EGL3 and the WD40 repeat protein TTG1 regulate the expression of *DFR, ANS, UFGT* and other downstream genes, affecting anthocyanin biosynthesis (Gonzalez et al., 2008; Saito et al., 2013). Recently, NAC (NAM, ATAF1,2, CUC2) transcription factors have also been reported to affect anthocyanin biosynthesis in blood-fleshed peaches (Zhou et al., 2015).

As one of the world's earliest cultivated fruit trees, more than 600 fig (*Ficus carica* L.) cultivars have been described (Flaishman et al., 2008). The fruit are termed syconia and demonstrate a typical double-sigmoid growth curve, including two rapid size-increment phases (phase I and III) and a slow growth phase between them (phase II) (Crane and Baker, 1953; Kislev and Bar-Yosef, 2006). When the fruit matures (in phase III), its colors are diverse; depending on the cultivar, the peel color can be green, yellow–green, yellow, red, purple, or violet–black. Fig peel color is primarily due to the accumulation of anthocyanins, with anthocyanin type and content differing among the different cultivars (Dueñas et al., 2008). Four anthocyanins have been reported in purple fig cultivars, namely cyanidin-3-O-glucoside, cyanidin-3-rutinoside, pelargonidin-3-glucoside and cyanidin-3,5-diglucoside. Yellow fig cultivars accumulate carotenoids such as lutein, zeaxanthin, β-cryptoxanthin and β-carotene (Yemiş et al., 2012). Cyanidin-3-O-glucoside chloride has been reported as the predominant anthocyanin in the peel of cvs. Black Mission and Brown Turkey (Solomon et al., 2006; Ercisli et al., 2012). As these trees rely mainly on vegetative propagation, mutation is an important agent of change in fig cultivar development.

“Green Peel” (“Qingpi”) is a major fig cultivar in China with green-colored fruit; “Purple Peel” (“Zibao”) is a bud mutation of “Green Peel,” with fruit that turn an appealing dark purple in phase III. In this study, targeted metabolome and transcriptome comparisons were carried out using young and mature fruit of “Green Peel” and “Purple Peel” fig. Beyond identifying specific anthocyanins in the mutant, we reveal very significant accumulation of a set of flavonoids and procyanidin B1, together with systematic transcriptional changes for structural genes, transcription factors and other regulators of the phenylpropanoid and flavonoid biosynthetic pathways, providing valuable information on fruit color and its complex effect on fruit quality components.

## Materials and methods

### Plant materials and treatments

The common fig cultivar Green Peel and its bud mutation cv. Purple Peel were cultivated in Weihai City (37°5′ N, 122°1′ W), Shandong Province in China. The soil type is sandy loam. The sampled fig orchard is 1 km from the sea and managed in the same way as the other orchards in this major fig-growing region in China. There were no significant or remarkable differences in 63 tested/observed morphological/horticultural items listed by the UPOV (International Union for the Protection of New Varieties of Plants, Geneva, Switzerland, http://upov.int) [UPOV TG/265/1 (E)] between “Green Peel” and its purple mutant, except for fruit color at ripening (Xu et al., 2016). The main crop fruit used for the metabolome study and RNA-sequencing (RNA-Seq) were collected on 18 Oct 2015, and fruit samples used for RT-qPCR validation were collected on 25 Oct 2016. The fig has a continuous fruiting characteristic, with different development stages of the main crop fruit growing along the shoots. Fruits in the late stage of phase II and in the middle of phase III were sampled from the two cultivars and termed “Green Peel” young fruit (GY), “Purple Peel” young fruit (PY), “Green Peel” mature fruit (GM) and “Purple Peel” mature fruit (PM), respectively. Three biological replicates were collected per sample, each with 20 fruits randomly collected from 15 fig trees in the same plot of the commercial orchard. We took the figs back to the laboratory, and the peel (about 2 mm thick) was carefully excised with a razor blade, collected, frozen in liquid nitrogen, roughly ground and kept at −80°C for further use.

### Extraction and separation of polyphenol secondary metabolites

Fig peel samples were further ground to a fine powder in liquid nitrogen and thoroughly mixed, then a ca. 3-g sample was freeze-dried and crushed using a mixer mill (MM 400, Retsch) with zirconia beads for 1.5 min at 30 Hz. Sample (100 mg) was extracted with 1 mL 70% methanol containing 0.1 mg/L lidocaine as an internal control for 12 h on a rotating wheel at 4°C in the dark. After 10,000 g centrifugation for 10 min at 4°C, the extracts were absorbed (CNWBOND Carbon-GCB SPE Cartridge, 250 mg, 3 mL; ANPEL, Shanghai, China, www.anpel.com.cn) and filtered (SCAA-104, 0.22-μm pore size; ANPEL) before LC–MS analysis. A quality-control sample was prepared by equal blending of all samples; during the assay, the quality control sample was run every 10 injections to monitor the stability of the analytical conditions.

Samples (5 μL) were injected into a HPLC system (Shim-pack UFLC SHIMADZU CBM30A) equipped with a C18 column (Waters ACQUITY UPLC HSS T3, 1.8 μm, 2.1 mm × 100 mm). The binary solvent system was ultra-pure water containing 0.04% acetic acid as mobile phase A and acetonitrile containing 0.04% acetic acid as mobile phase B. The A:B (v/v) gradient was 95:5 at 0 min, 5:95 at 11.0 min, 5:95 at 12.0 min, 95:5 at 12.1 min, 95:5 at 15.0 min. The flow rate was kept at 0.40 mL/min, and the column temperature was maintained at 40°C.

### Metabolite identification and quantification

The HPLC effluent was connected to an electrospray ionization (ESI)-triple quadrupole-linear ion trap–MS/MS system (Applied Biosystems 4500 Q TRAP). Metabolite identification and quantification were carried out following Chen et al. (2013). In brief, the inspected mass spectra were 50–1,000 m/z. Nitrogen was used as both the nebulizer/auxiliary and collision gas. The ESI source was set to positive ionization mode, the source temperature was held at 550°C; the capillary voltage was 5.5 kV. The monitoring mode was set to multiple-reaction monitoring (MRM).

Metabolite identification was based on the primary and secondary spectral data annotated against public databases, namely MassBank (http://www.massbank.jp/), KNAPSAcK (http://kanaya.naist.jp/KNApSAcK/), HMDB (http://www.hmdb.ca/), MoToDB (http://www.ab.wur.nl/moto/), and METLIN (http://metlin.scripps.edu/index.php), following the standard metabolic operating procedures. Metabolite quantification was carried out using MRM. Partial least squares discriminant analysis (PLS–DA) was carried out with the identified metabolites. Metabolites with significant differences in content were set with thresholds of variable importance in projection (VIP) ≥ 1 and fold change ≥ 2 or ≤ 0.5.

### RNA-Seq and annotation

RNA isolation and purification, and cDNA library construction and sequencing were as performed previously (Chai et al., 2017). In brief, fig samples' total RNA was extracted by the CTAB method (Cao et al., 2016). RNA quantity and quality were determined by NanoDrop ND1000 spectrophotometer (NanoDrop Technologies, Wilmington, DE, USA) and the Agilent Bioanalyzer 2100 system (Agilent Technologies, Palo Alto, CA, USA), respectively. RNA integrity was determined by 1% agarose gel electrophoresis, and the RNA concentration was adjusted for uniformity. mRNA was isolated from total RNA using magnetic beads with oligo (dT); cDNA was synthesized using a cDNA Synthesis Kit (TaKaRa) and linking the sequencing adapter to both ends (Chai et al., 2014). The library preparations were sequenced on an Illumina HiSeq 4000 platform and the unigene sequences obtained from our laboratory transcriptome database by RSEM software were integrated for annotation (Chai et al., 2017). The whole set of annotated genes can be found in the National Center for Biotechnology Information (NCBI) SRA database (accession number SRP114533).

### Analysis of differentially expressed genes (DEGs)

For gene-expression analysis, counts were mapped to the reading of each gene by HTSeq v0.5.4p3 and then normalized to FPKM (fragments per kilobase of transcript per million mapped reads) following Mao et al. (2005). DEGs were recruited by log_2_ (fold change) ≥ 1 and corrected *P* ≤ 0.005. All DEGs were analyzed by gene ontology (GO) enrichment using GOseq (1.10.0) (Götz et al., 2008) and Kyoto Encyclopedia of Genes and Genomes (KEGG) enrichment using KOBAS software (Mortazavi et al., 2008).

### Real-time quantitative PCR (RT-qPCR) validation

RNA extraction and quality detection were carried out by RNA-Seq. Reverse transcription was performed using HiFi-MMLV cDNA First-Strand Synthesis Kit (Invitrogen). Twenty color-related genes were selected for RT-qPCR with specific primers designed by Primer Premier 5 software (Supplementary Table 1). The RT-qPCR was performed with an ABI 7500 Fast Real-Time Detection System (Applied Biosystems) using the Ultra SYBR Mix kit (CWBIO, Beijing, China). The amplification system consisted of 10.4 μL Ultra SYBR Premix System II, 0.8 μL of 10 μmol/L upstream primer, 0.8 μL of 10 μmol/L downstream primer, 2 μL template, and sterile distilled water to a total volume of 20 μL. The amplification program was 95°C for 10 min, followed by 40 cycles of 95°C for 15 s and 55°C for 1 min. Relative quantitative analysis of data was performed by the 2^−ΔΔCT^ method with reference genes β*-actin* and *18S-RNA*. Three technical replicates were carried out for each sample to ensure reproducibility and reliability. Statistical analysis of variance (ANOVA) followed by Duncan's new multiple range test were performed with SPSS Version 16.0 (Chicago, IL, USA). The significance level was set to *P* < 0.05.

## Results

### Phenotype of “green peel” and its mutant “purple peel”

**Figure 1:**
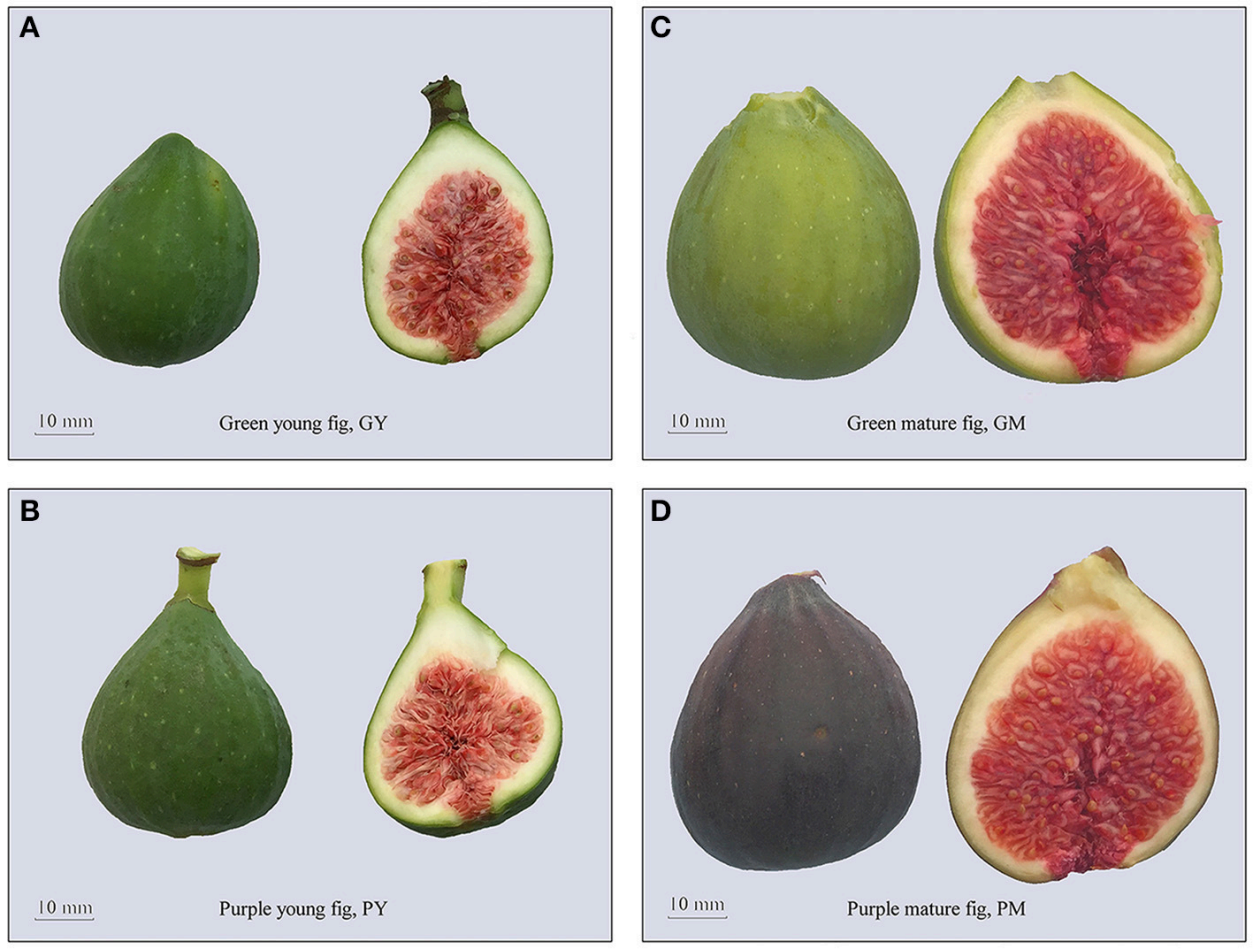
The phenotype of fig (*Ficus carica* L.) cv. Green Peel and its mutation cv. Purple Peel at young and mature stages. **(A)** “Green Peel” young fruit. **(B)** “Purple Peel” young fruit. **(C)** “Green Peel” mature fruit. **(D)** “Purple Peel” mature fruit. GY, “Green Peel” young fruit; GM, “Green Peel” mature fruit; PY, “Purple Peel” young fruit; PM, “Purple Peel” mature fruit.

No morphological differences were detected between the fruit of “Green Peel” and its purple mutant, except for fruit color at ripening. The young fruit used in the present study were harvested in the late stage of fig development phase II, when both “Green Peel” and its purple mutant have a deep green appearance, with a very slight copper hue on the surface of the purple mutant. When the fruits were halved, the texture was hard, and the internal female flowers were a pink–garnet color (Figures 1A,B).

Fig development is very rapid in phase III. The fruit quickly enlarge, reaching their final size and harvest quality in 5–7 days. “Green Peel” fruit turned yellow–green in appearance, whereas the mutant developed a dark purple peel. Mature fruit were soft and succulent, and female flowers of both cultivars were deep red inside the fruit (Figures 1C,D). As a measure of fruit quality, “Green Peel” and its purple mutant had an average fruit weight of 33.9 ± 2.66 g and 33.4 ± 2.4 g, 18.51 ± 1.03 and 18.34 ± 1.15 °Brix in soluble solids, and peel thickness of 2.14 ± 0.32 and 2.16 ± 0.24 mm, respectively, with no significant differences in the assayed horticultural attributes.

### Targeted secondary metabolite assay

The general secondary metabolite profiles of “Green Peel” and “Purple Peel” fig fruit showed marked differences (Supplementary Figures 1, 2). A total of 101 metabolites were identified from GY, PY, GM, and PM samples, each with three biological replicates: 18 phenylpropanoids, 40 flavones, 12 flavonols, 16 flavonoids, 8 anthocyanins, 5 proanthocyanidins, and 2 catechin derivatives (Table 1). Setting VIP ≥ 1.0 together with fold change ≥ 2 or ≤ 0.5 as thresholds for significant differences, the contents of 5 and 22 metabolites were significantly different between “Green Peel” and its purple mutant at the young and mature stage, respectively.

**Table 1:** Differentially accumulated phenolic compounds in the peel of “Green Peel” and “Purple Peel” fruit.

**Component name**	**Metabolite name**	**Content**		**Fold change (PY/GY; PM/GM)**	**VIP**
		**Green fig**	**Purple fig**		
**ANTHOCYANIN**					
GY vs. PY	Cyanidin O-malonylhexoside	3.81E + 03	2.68E + 04	7.03	3.00541
	Cyanidin O-malonylhexoside O-hexoside	6.37E+03	1.85E+04	2.90	2.21738
GM vs. PM	Cyanidin O-malonylhexoside	1.93E+03	7.69E+06	3992.21	3.42056
	Cyanidin 3-O-glucoside	1.38E+05	6.37E+07	461.40	2.96158
	Cyanidin O-malonylhexoside O-hexoside	5.85E+03	2.44E+06	416.60	2.91667
	Cyanidin-3,5-O-diglucoside (cyanin)	4.62E+05	5.26E+07	113.87	2.58938
**PROCYANIDIN**					
GM vs. PM	Procyanidin B1	2.79E+04	8.98E+06	322.26	2.84928
	Procyanidin B2	3.64E+04	1.67E+05	4.58	1.44844
	Procyanidin B	1.30E+04	4.69E+04	4.02	1.29685
	Procyanidin B3	3.03E+03	1.22E+04	3.60	1.32665
	Procyanidin A	8.23E+03	1.77E+04	2.15	1.01483
**FLAVONE**					
GY vs. PY	Apigenin	5.67E+04	2.37E+04	0.42	1.90703
GM vs. PM	Luteolin-3′,7-di-O-glucoside	1.98E+05	1.11E+07	56.06	2.38774
	3′,6-Dimethylflavone	7.24E+03	1.77E+04	2.44	1.02284
	Chrysin	1.22E+05	5.19E+04	0.43	1.09238
	Tangeretin	4.13E+04	1.28E+04	0.31	1.28301
**FLAVONOIDS**					
GY vs. PY	7-O-Methyleriodictyol	1.91E+04	6.37E+03	0.33	2.18819
GM vs. PM	Epicatechin (EC)	6.22E+04	8.15E+05	13.09	1.87203
	Catechin (C)	5.95E+04	3.32E+05	5.57	1.52977
	Hesperetin 5-O-glucoside	2.32E+06	5.91E+06	2.55	1.13184
	7-O-Methyleriodictyol	8.73E+03	1.84E+04	2.10	1.02489
**FLAVONOL**					
GM vs. PM	Quercetin-3-O-α-arabinofuranoside (Avicularin)	2.60E+04	9.77E+04	3.76	1.32249
	Quercetin-3-O-glucoside (isoquercitrin)	6.44E+06	1.50E+07	2.33	1.08046
**PHENYLPROPANOIDS**					
GY vs. PY	Esculetin	6.67E+03	1.37E+04	2.05	1.75354
GM vs. PM	Quinic acid	5.90E+03	2.55E+04	4.32	1.39172
	Cinnamic acid	2.09E+05	6.99E+04	0.34	1.19368
	Esculetin	1.16E+04	2.40E+03	0.21	1.4841

*GY, “Green Peel” young fruit; PY, “Purple Peel” young fruit; GM, “Green Peel” mature fruit; PM, “Purple Peel” mature fruit. Metabolite fold changes, value >1.0 represents increase; value < 1.0 represents decrease. Differentially accumulated phenolic compounds were identified by threshold VIP (variable importance in projection) ≥1, and fold change ≥2 (upregulation) or ≤ 0.5 (downregulation)*.

### Anthocyanins

Four kinds of cyanidin glycosides, delphinidin O-hexoside, malvidin-3-O-galactoside and rosinidin O-hexoside were identified in all samples. In the PY peel, cyanidin O-malonylhexoside and cyanidin O-malonylhexoside O-hexoside were found with 7.03- and 2.9-fold increments compared to GY, which could explain the slight hue on the PY peel. At the mature stage, cyanidin glucoside pigments were responsible for the mutant purple color: cyanidin O-malonylhexoside was increased 3,992.21-fold in the PM vs. GM samples, whereas cyanidin 3-O-glucoside, cyanidin O-malonylhexoside O-hexoside and cyanidin-3,5-O-diglucoside increased 461.4-, 416.6-, and 113.87-fold, respectively (Table 1).

### Flavonoids, flavones, and flavonols

Among the monomeric flavonoids, epicatechin, catechin, hesperetin 5-O-glucoside, and 7-O-methyleriodictyol demonstrated significantly higher contents in the PM; epicatechin was 13.09-fold its content in GM. In young fig fruit samples, apigenin and flavanone 7-O-methyleriodictyol showed 1.2- and 1.6-fold decreases in GY vs. PY (Table 1), but no other differences met the criteria.

The A- and B-type procyanidins are dimer flavonoids; their contents only differed in the mature fruit group. The content of procyanidin B1 [epicatechin-(4β → 8)-catechin] was 322.26-fold higher in the GM vs. PM fruit. Procyanidins B2 [(–)-Epicatechin-(4β → 8)-(–)-epicatechin], B3 [catechin-(4α → 8)-catechin], A1 [epicatechin-(2β → 7,4β → 8)-catechin] and A2 [epicatechin-(2β → 7,4β → 8)-epicatechin] were 2- to 4.5-fold higher in the GM vs. PM (Table 1), which were much less than that of procyanidin B1 in the fruit.

For the flavones, luteolin-3′,7-di-O-glucoside and 3′6-dimethylfavone contents were 56.06- and 2.44-fold higher, respectively, in the GM vs. PM. Chrysin and tangeretin revealed significant decreases in the PM, whereas apigenin, the upstream substrate of luteolin, was remarkably lower in the GY vs. PY. A significant increase was found for two quercetin glycosides in the GY vs. PY with a moderate fold change (Table 1).

### Phenylpropanoids

The phenylpropanoid biosynthetic pathway is upstream of the anthocyanin and flavonoid biosynthetic pathways. We identified 18 general metabolites of phenylpropanoids. Esculetin and quinic acid contents were 2.05- and 4.33-fold higher in the PY and PM peels, respectively, whereas cinnamic acid and esculetin contents in the PM were less than half that in the GM (Table 1).

### Transcriptome analysis

RNA-Seq produced 31,591,009, 25,146,641, 32,429,280 and 27,147,120 clean reads from GY, PY, GM and PM libraries, respectively. Clean data from the 12 libraries of 4 samples (3 replicates for each samples), were averaged to 96,158 transcripts of 796.42 bp in length, and 79,355 unigenes were obtained using Trinity software (Supplementary Table 2). The N50 value was 1236 bp, and the average length of the unigenes was 683.07 bp.

**Figure 2:**
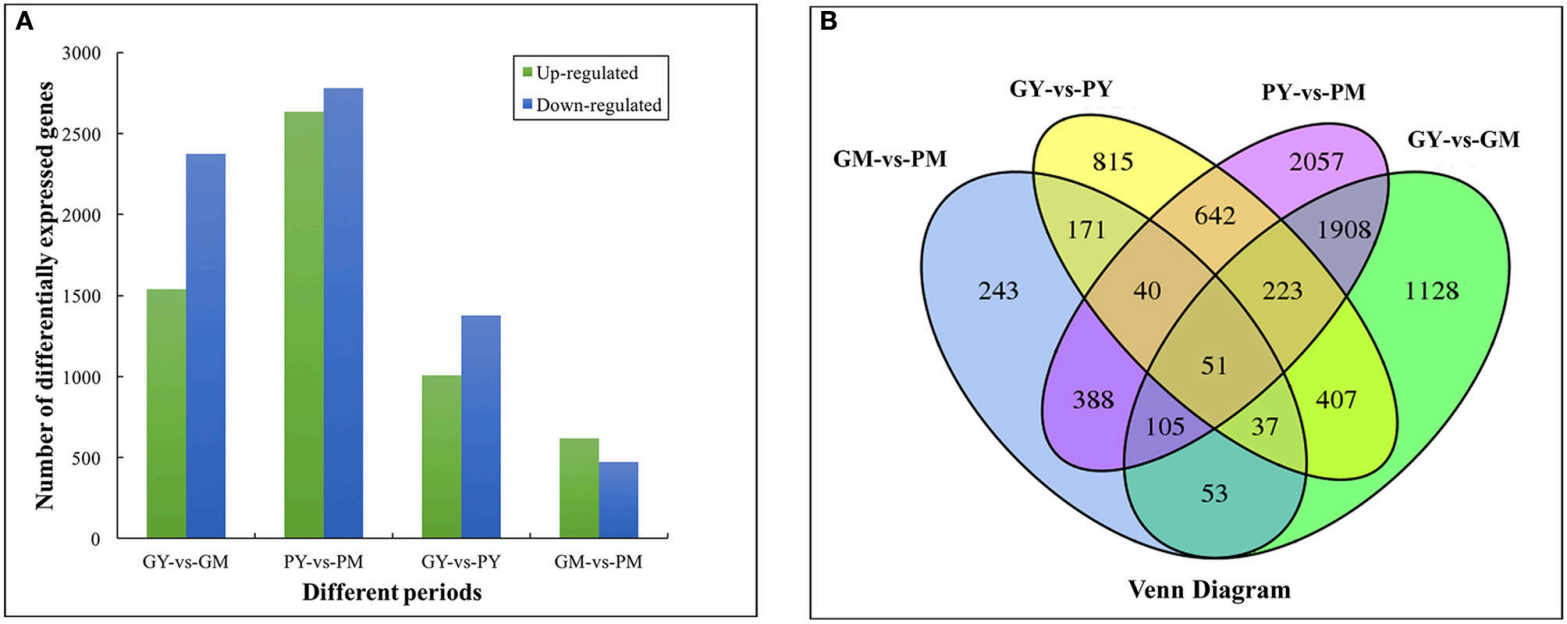
Functional annotation and classification of differentially expressed genes between young and mature stages of “Green Peel” and “Purple Peel.” **(A)** Numbers of differentially expressed genes. **(B)** Venn diagram. GY, “Green Peel” young fruit; GM, “Green Peel” mature fruit; PY, “Purple Peel” young fruit; PM, “Purple Peel” mature fruit.

There were 2,385, 1,087, 3,911, and 5,413 DEGs in the four comparison groups: GY vs. PY, GM vs. PM, GY vs. GM, and PY vs. PM, respectively. Comparing the two cultivars, 1,009 and 616 genes were upregulated, and 1,376 and 471 genes were downregulated in GY vs. PY and GM vs. PM, respectively (Figure 2A). Venn diagram analysis showed 51 DEGs that were common to all four comparison groups (Figure 2B). GO analysis assigned 46,748, 34,527 and 22,307 unigenes to the biological process, cell component and molecular functional class, respectively (Supplementary Figure 3). The clusters of orthologous groups of proteins database (COG) annotation allocated 15,726 unigenes into 25 COG categories (Supplementary Figure 4); the general functional cluster prediction (2,115 unigenes, 13.45%) was the largest group, followed by signaling mechanism (1,897 unigenes, 12.06%), posttranslational modification and protein turnover (1,572 unigenes, 10.00%).

KEGG analysis revealed plant hormone signal transduction, starch and sucrose, phenylpropanoid biosynthesis and alpha-linolenic acid metabolism as the significantly changed pathways in GY vs. PY. Plant hormone signal transduction, phenylpropanoid and flavonoid biosynthetic pathways were significantly changed in GY vs. GM and GM vs. PM (Table 2).

**Table 2:** Significantly enriched KEGG pathways between “Purple Peel” and “Green Peel” figs.

**No**.	**Pathway**	**DEGs with pathway annotation**	**All genes with pathway annotation**	***P*-value**	**Corrected *P*-value**	**Pathway ID**
**GY vs. PY**						
1	Plant hormone signal transduction	34	227	1.96E-11	6.13E-09	ko04075
2	Starch and sucrose metabolism	22	281	5.22E-07	8.17E-05	ko00500
3	Phenylpropanoid biosynthesis	3	182	3.38E-05	0.003522746	ko00940
4	Alpha-linolenic acid metabolism	14	66	8.31E-05	0.006499291	ko00592
**GM vs. PM**						
1	Flavonoid biosynthesis	17	52	4.02E-08	1.06E-05	ko00941
2	Protein processing in endoplasmic reticulum	32	318	1.34E-06	0.000176186	ko04141
3	Estrogen signaling pathway	16	92	3.99E-06	0.000349801	ko04915
**GY vs. GM**						
1	Plant hormone signal transduction	58	227	9.51E-16	3.03E-13	ko04075
2	Phenylpropanoid biosynthesis	10	182	8.45E-09	1.35E-06	ko00940
3	Flavonoid biosynthesis	15	52	3.71E-07	3.95E-05	ko00941
**PY vs. PM**						
1	Plant hormone signal transduction	62	227	1.36E-10	4.45E-08	ko04075
2	Flavonoid biosynthesis	23	52	4.44E-07	7.26E-05	ko00941
3	Phenylpropanoid biosynthesis	11	182	1.39E-05	0.001513077	ko00940

*GY, “Green Peel” young fruit; PY, “Purple Peel” young fruit; GM, “Green Peel” mature fruit; PM, “Purple Peel” mature fruit. Significant pathways were identified by corrected P ≤ 0.01*.

### Phenylpropanoid, flavonoid, and anthocyanidin biosynthetic pathways

**Figure 3:**
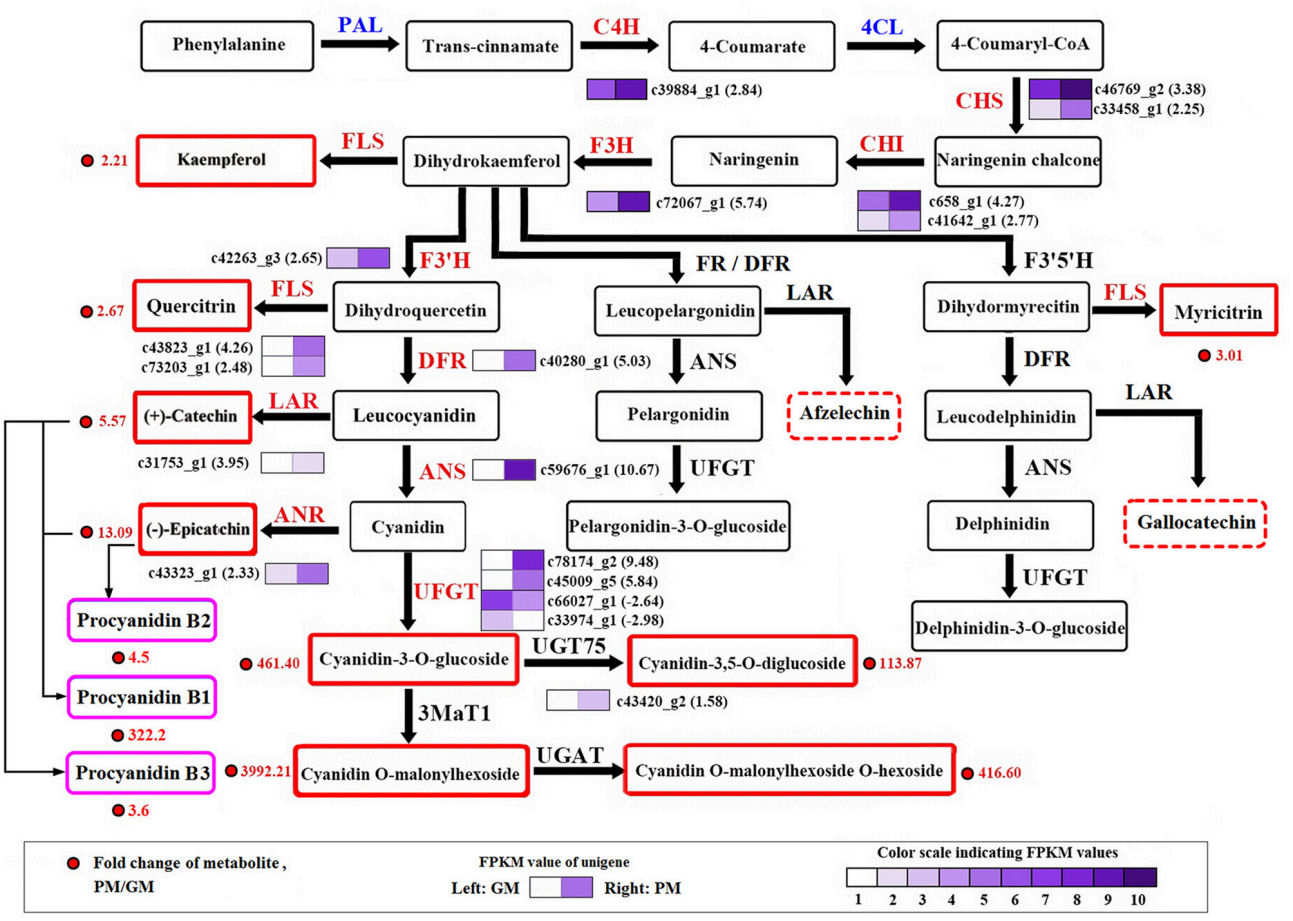
Transcript profiling of genes in the phenylpropanoid and flavonoid biosynthetic pathways in cv. “Green Peel” and “Purple Peel” at mature stages. GM, “Green Peel” mature fruit; PM, “Purple Peel” mature fruit. Grids with color-scale from light to dark represent RPKM values 0–10, 10–20, 20–40, 40–80, 80–160, 160–320, 320–640, 640–1,280, 1,280–2,560, and over 2,560, respectively. PAL, phenylalanine ammonia-lyase; C4H, cinnamic acid 4-hydroxylase; 4CL, 4-coumarate CoA ligase; CHS, chalcone synthase; CHI, chalcone isomerase; F3H, flavanone 3-hydroxylase; F3′H, flavanoid 3′-hydroxylase; DFR, dihydroflavonol 4-reductase; FR, flavanone 4-reductase; ANS/LDOX, anthocyanidin synthase/leucocyanidin oxygenase; UFGT, UDP glucose-flavonoid 3-O-glcosyl-transferase; FLS, flavonol synthesis; LAR, leucocyanidin reductase; ANR, anthocyanin reductase.

At maturity, most of the secondary metabolite pathways were strengthened by gene-expression upregulation in the “Purple Peel” mutant fruit, except for the DEGs *PAL* and *4CL*. Two *PAL* genes (*c388_g1* and *c388_g2*) were downregulated (-1.14- and−1.02-fold) and five *4CL* unigenes were downregulated, in line with the decreased cinnamic acid content in the PM peel. Simultaneous large-scale upregulation of structural genes of the phenylpropanoid, flavonoid and anthocyanin biosynthetic pathways, including *CHS, CHI*, and *flavonol synthase* (2 DEGs each), *UFGT* (4 DEGs), and other genes (1 DEG) dominated secondary metabolite synthesis modulation in GM vs. PM (Figure 3). High fold upregulation and high RPKM (reads per kilobase of transcript per million mapped reads) enhanced the flux in the flavonoid and anthocyanidin biosynthetic pathways. Structural genes *CHS* (*c46769_g2*) and *CHI* (*c658_g1*) showed 3.38- and 4.27-fold increments, *F3H* (*c72067_g1*) 5.47-fold upregulation, *F3*′*H* (*c42263_g3*) 2.65-fold upregulation, together with 2 flavonol synthase genes that not only catalyze the conversion from kaempferol to quercetin (Pelletier et al., 1997), but also from apigenin to luteolin (Martens et al., 2003; Jaakola, 2013); this could largely explain the high accumulation of luteolin-3′,7-di-O-glucoside in the PM (Table 1). Catechin is produced from leucocyanidin catalyzed by leucoanthocyanidin reductase (LAR) (*c31753_g1*, 3.95-fold upregulation); the enzyme also catalyzes leucodelphinidin and leucopelargonidin to gallocatechin and afzelechin, respectively, neither of which demonstrated significant content differences between the cultivars, corresponding to the lower change in content of A-type procyanidin (Table 1, Figure 3).

*LAR* expression (*c31753_g1*) was upregulated 3.95-fold. *ANS* (*c59676_g1*) was one of the most significantly DEGs in the GM vs. PM group, increasing 10.67-fold, followed by two *UFGT* genes (*c78174_g2* and *c45009_g5*) which showed 9.98-fold and 5.84-fold upregulation in the PM fruit (Figure 3). Anthocyanidin 3-O-glucosyltransferase 2 (*c45009_g5*, 5.84-fold upregulation) catalyzes cyanidin to cyanidin-3-O-glucoside. Cyanidin-3,5-O-diglucoside can be glycosylated from cyanidin-3-O-glucoside or cyanidin-5-O-glucoside; UDP-glycosyltransferase 75D1 (*c43420_g2*, 1.58-fold upregulation) catalyzes cyanidin-3-O-glucoside to cyanidin-3,5-O-diglucoside, which also supports the high measured accumulation of the two cyanidin mono- and di-glucosides, flavonoids and procyanidins in the “Purple Peel” fig (Table 1, Figure 3).

### Transcription factors

There were 74 and 45 differentially expressed transcription factor genes identified in GY vs. PY and GM vs. PM, respectively, whereas from young fruit to mature fruit, 140 and 141 DEGs were identified as transcription factors in GY vs. GM and PY vs. PM, respectively (Table 3, Supplementary Table 3). The differentially expressed transcription factors were annotated as encoding MYB, bHLH, AP2/ERF, WRKY, HD-ZIP, heat-shock transcription factor (HSF), NF-Y, DIVARICATA, and MADS-box (Table 3).

**Table 3:** Differentially expressed transcription factors in the peel of young and mature fruit of “Green Peel” and “Purple Peel” fig.

**Comparison group**	**Gene name**	**Number of DEGs**	**Upreg-ulated DEGs**	**Downreg-ulated DEGs**	**Description**	**Biological functions**
GY vs. PY	MYB	19	6	13	MYB TFs	Cell development and anthocyanin pathway
	AP2/ERF	21	6	15	Ethylene-responsive TF	Plant development and stress response
	bHLH	13	2	11	Basic helix-loop-helix protein	Plant development and substance metabolism
	Other TFs	21	13	8		
	In total	74	27	47		
GM vs. PM	MYB	9	5	4	MYB TFs	Cell development and anthocyanin pathway
	AP2/ERF	10	10	0	Ethylene-responsive TF	Plant development and stress response
	bHLH	8	2	6	Basic helix-loop-helix protein	Plant development and substance metabolism
	Other TFs	18	6	7		
	In total	45	23	17		
GY vs. GM	bHLH	29	4	25	Basic helix-loop-helix protein	Plant development and substance metabolism
	MYB	33	7	26	MYB TFs	Cell development and anthocyanin pathway
	AP2/ERF	22	11	11	Ethylene-responsive TF	Plant development and stress response
	WRKY	18	9	9	WRKY DNA-binding protein	Defense responses and plant development
	HD-ZIP	8	3	5	Homeobox-leucine zipper protein	Photomorphogenesis and fruit ripening
	MADS-box	5	0	5	MADS-box TFs	Fruit development and ripening
	Other TFs	25	1	24		
	In total	140	35	105		
PY vs. PM	MYB	29	18	11	MYB TFs	Cell development and anthocyanin pathway
	bHLH	26	7	19	Basic helix-loop-helix protein	Plant development and substance metabolism
	AP2/ERF	19	12	7	Ethylene-responsive TF	Plant development and stress response
	WRKY	15	11	4	WRKY DNA-binding protein	Defense responses and plant development
	HD-ZIP	10	0	10	Homeobox-leucine zipper protein	Photomorphogenesis and fruit ripening
	HSF	8	7	1	Ethylene-responsive TF	Plant growth, development and stress response
	HAP	4	0	4	Nuclear TF Y subunit A	Embryonic development and chloroplast biogenesis
	Other TFs	30	11	19		
	In total	141	66	75		

*GY, “Green Peel” young fruit; PY, “Purple Peel” young fruit; GM, “Green Peel” mature fruit; PM, “Purple Peel” mature fruit. Differentially expressed genes were identified by FDR ≤ 0.001 and absolute value of log_2_ ratio ≥ 2*.

**Figure 4:**
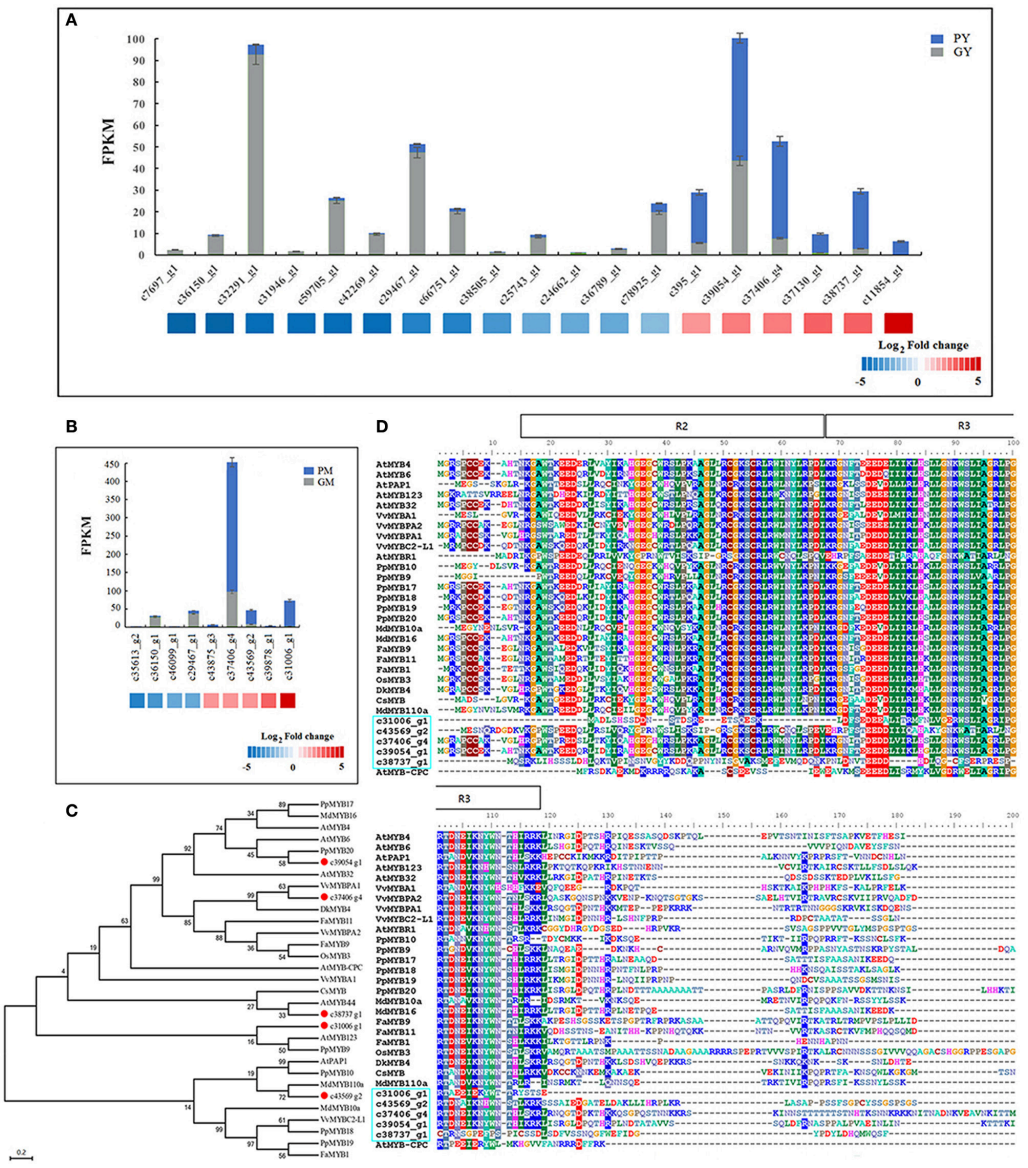
Differentially expressed *MYB* genes between “Green Peel” and “Purple Peel” fruit at young and mature stages. **(A)** Differentially expressed *MYB* genes between the two cultivars' young fruit. **(B)** Differentially expressed *MYB* genes between the two cultivars' mature fruit. **(C)** Phylogenetic analysis of five fig *MYBs* recruited by high fold expression change. **(D)** R2R3-MYB protein sequence alignment of five fig *MYBs* recruited by high fold expression change; R2R3 motif is indicated at the top.

Almost all of the *MYB* DEGs could be further assigned to the *R2R3 MYB* family, which is closely associated with anthocyanin biosynthesis in fruit trees (Allan et al., 2008; Liu et al., 2016). Nineteen and nine *R2R3-MYB*s were differentially expressed in the young fruit (GY vs. PY) and mature fruit (GM vs. PM), respectively. Among the *MYB* DEGs in young fruit, 6 genes were found upregulated and 13 downregulated in PY (Figure 4A). In mature fruit, there were 9 recognized *MYB* DEGs: 5 more highly expressed *MYB*s in PM, and 4 more highly expressed *MYB*s in GM, but all with low FPKM values (Figure 4B). Along fig fruit development, 33 *MYB* DEGs (7 upregulated and 26 downregulated) were illustrated in GY vs. GM, 29 *MYB* DEGs (18 upregulated and 11 downregulated) in PY vs. PM (Table 3). Nine *MYB*s had significantly increased transcripts in both PY vs. PM and GM vs. PM, 4 of them also showing upregulation in GY vs. GM.

We further recruited five *R2R3-MYB*s—unigenes c*31006*_g1, *c39054_g1, c37406_g4, c38737_g1*, and *c43569_g2*—which showed high fold change in expression between the two cultivars and/or developmental stages (Figures 4A,B). The expression of *c43569_g1* and *c31006_g1* was specifically increased in PM. Protein sequence comparison revealed that c43569_g1 is highly homologous (72%) to MdMYB110a of apple (Figure 4C), which plays a key role in the red flesh apple phenotype (Chagné et al., 2013). The unigene c31006_g1 clustered with AtMYB123 of *Arabidopsis* and PpMYB9 of *Prunus persica*, which regulates anthocyanin accumulation in different plant tissues (Zhou et al., 2016). The highly expressed MYB c39054_g1 in PY was closely related to the flavonoid MYB repressor PpMYB20 (Figure 4C; Zhou et al., 2016), whereas c37406_g4 and c38737_g1 clustered with the anthocyanin activator groups, with high similarity to AtMYB44, VvMYBPA1 and VvMYBPA2, which regulate anthocyanin biosynthesis in *Arabidopsis* and grape (Terrier et al., 2009; Jung et al., 2010; Zhou et al., 2016). Figure 4D illustrates the fig R2R3-MYBs' highly homologous R2 and R3 DNA-binding domains at the N-terminus (Espley et al., 2007), and highly variable truncated C-terminal region, which might relate to fig color morph regulation.

Thirteen *bHLH* DEGs were found in young fruit (GY vs. PY); 2 were highly expressed in PY, and 11 were downregulated. Eight *bHLH* revealed differences in mature-stage fruit (GM vs. PM): 2 *bHLH* were highly expressed in PM (Table 3). During fruit development, 29 *bHLH* DEGs were screened in the Green Peel cultivar, including 4 upregulated and 25 downregulated from young to mature fruit, whereas among 26 *bHLH* of the Purple Peel cultivar, 7 contigs or transcripts were upregulated and 19 were downregulated from young to mature fruit (Table 3). We found 2 *bHLH* DEGs (FPKM ≥ 300)—*c21697_g1* and *c43844_g1*—expressed at very high levels in the PY, that decreased rapidly at the mature stage of “Purple Peel,” and their expression levels were very low in GY and GM (Supplementary Figure 5).

### Heat-shock proteins (HSPs)

HSPs are involved in protein synthesis, folding, cell localization and protein degradation; they also play a role in maintaining intercellular environmental stability (Wang et al., 2004; Waters, 2013). In the mature fig fruit, 15 small HSP family DEGs were identified, including 9 *HSP20*, 3 *HSP90*, 2 *HSP70*, and 1 *HSP40*, all of which showed significantly higher expression in the GM vs. PM (Table 4); moreover, 3 genes encoding heat-shock transcription factors (HSFs) (*c45384_g1, c26517_g2*, and *c43194_g3*) showed a significant expression increment in the PM (Table 4). HSFs bind to the heat shock element of the HSP gene promoter to form transcription complexes, which promote HSP gene expression (Scharf et al., 2012). HSPs are molecular chaperones, also known as stress-induced proteins, which function in protein folding and assembly, protect enzymes from denaturation and cellular degeneration with pigment and flavonoid accumulation, responding to stress and maturation in fig (Sun et al., 2002; Neta-Sharir et al., 2005).

**Table 4:** Differentially expressed heat-shock protein (HSP) and heat-shock transcription factor (HSF) genes in the mature stage of “Purple Peel” and “Green Peel” fig.

**Gene name**	**Seq_ID**	**Log_2_ FC (PM/GM)**	***P*-value**	**GM_ FPKM**	**PM_ FPKM**	**Regulated**
HSP20	c44815_g2	5	1.03E-06	1.92	64.35	Up
	c46276_g2	4.39	1.01E-15	33.21	698.96	Up
	c32064_g1	4.22	5.79E-38	16.06	301.27	Up
	c46276_g3	4.08	1.99E-33	34.29	582.76	Up
	c46276_g1	3.08	2.27E-32	52.55	445.82	Up
	c44815_g1	3.07	1.52E-25	26.18	220.94	Up
	c22071_g1	2.71	6.01E-07	3.02	20.29	Up
	c46998_g1	2.69	2.08E-15	5.43	35.67	Up
	c25561_g1	2.65	3.11E-12	4.63	29.67	Up
HSP70	c43747_g1	7.19	5.85E-06	0	14.5	Up
	c46871_g7	6.83	1.25E-32	0	11.25	Up
	c45569_g1	3.57	9.76E-30	2.11	26.18	Up
HSP90A	c39629_g1	4.69	3.12E-10	0	2.47	Up
	c31839_g1	3.99	8.83E-08	6.36	102.47	Up
HSP40	c39984_g1	3.83	8.29E-43	14.91	213.78	Up
HSF	c43194_g3	3.75	3.96E-41	6.35	86.71	Up
	c26517_g2	3.05	2.30E-04	1.69	14.73	Up
	c45384_g1	2.2	4.22E-12	7.3	33.91	Up

*Differentially expressed genes were identified by FDR ≤ 0.001 and absolute value of log_2_ ratio ≥ 2 (2-fold). FC, fold change*.

### RT-qPCR validation of the transcriptomic data

**Figure 5:**
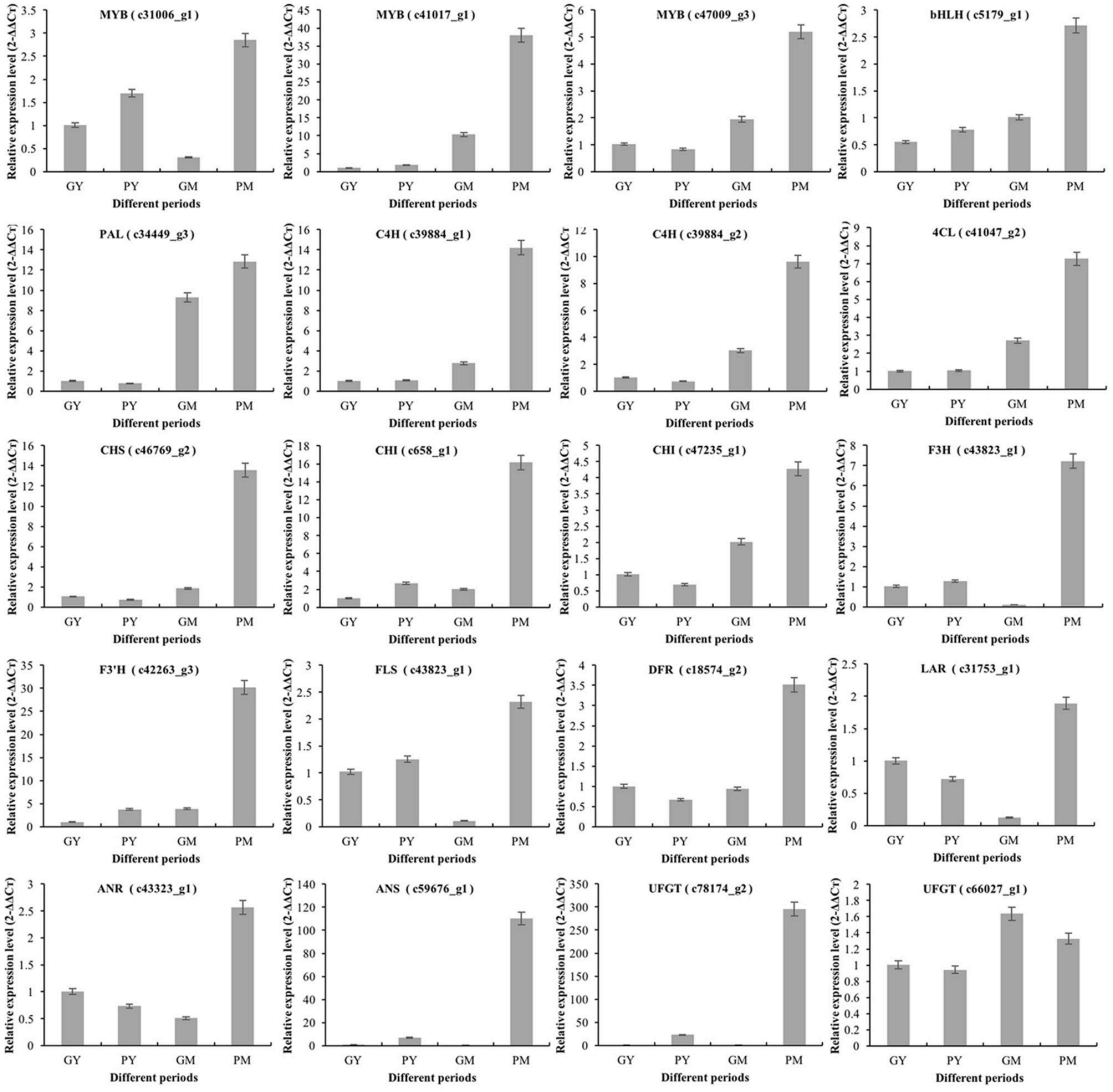
Expression of representative genes in young and mature stages of “Purple Peel” and “Green Peel” fig fruit validated by qRT-PCR. GY, “Green Peel” young fruit; GM, “Green Peel” mature fruit; PY, “Purple Peel” young fruit; PM, “Purple Peel” mature fruit.

To validate the key RNA-Seq results, we selected 20 DEGs (4 transcription factor genes, 4 phenylpropanoid biosynthetic pathway genes, and 12 flavonoid biosynthetic pathway genes) (Supplementary Figure 6) and analyzed their expression levels in PY, GY, PM, and GM using RT-qPCR. The expression patterns of these genes were very similar to the RNA-Seq results, with correlation coefficients (*R*^2^) > 0.83 (Figure 5). The results validated the relevance of the RNA-Seq data and RT-qPCR showed good consistency for both up- and downregulated gene expression.

## Discussion

Natural mutations have been, and still are, observed, deliberately selected for and used in fruit crop production. However, the resultant differences in gene structure and expression regulation in the mutants has only recently begun to be revealed. A combined metabolome and transcriptome study can provide us with new, large-scale information on the shifted secondary metabolic product profiles and the underlying modifications in gene-expression networks.

### Large-scale secondary metabolite and pathway regulation

Color mutants are widely used in horticultural and other crops, especially those that are commonly propagated vegetatively, such as most fruit trees. The color mutants are usually promoted and regarded as presenting a single-attribute difference. Herein, we identified 4 cyanidin glycosides in “Purple Peel” fig fruit, determined the substance responsible for the mutated purple color, and more importantly, revealed highly significant accumulation of colorless procyanidin B1, luteolin-3′,7-di-O-glucoside, epicatechin and other important secondary metabolites in the phenylpropanoid and flavonoid biosynthetic pathways. These findings illustrate, for the first time, a panorama of the large-scale secondary metabolite changes for a color mutation in the ancient fruit crop *Ficus carica*. The cyanidin glucosides in PM differed from those in other dark-colored fig cultivars, such as cyanidin-3-O-rhamnoglucoside (cyanidin-3-O-rutinoside), reported as the main anthocyanin in the peel of “Black Mission,” “Bursa,” and “Brown Turkey” figs (Solomon et al., 2006; Ercisli et al., 2012). Acyl-modified anthocyanins are common in *Arabidopsis* (D'Auria et al., 2007), and increased cyanidin 3-O-(malonyl)-glucoside has been reported in the cool-cultivated red lettuce to be the only pigment responding to temperature (Becker et al., 2014). A comparison of different cranberry cultivars indicated that highly pigmented berries also have higher contents of colorless flavonol (Bilyk and Sapers, 1986). Anthocyanins and flavonoids affect fruit color and taste; their antioxidant and nutraceutical capacities confer healthful properties, reducing the risk of cardiovascular morbidity and mortality (Holt et al., 2002; Wu et al., 2012).

The large-scale transcription expression increments in phenylpropanoid and flavonoid biosynthetic pathway genes in “Purple Peel” fig, revealed by RNA-Seq, strongly supported our metabolome results. Similarly, most of the structural genes in the anthocyanin biosynthetic pathway are upregulated during fruit development of red vs. green color mutations of pear (Yang et al., 2013). Coordinated expression changes of *F3H, F3*′*H, DFR1, ANS*, and *UFGT* have also been demonstrated in differently colored Chinese bayberries (Niu et al., 2010), grapes (Boss et al., 1996), *Arabidopsis* (Pelletier et al., 1997; Saito et al., 2013) and other plants (Quattrocchio et al., 1993).

The mutated color attribute is observed late in fruit development. However, significant changes in phenylpropanoid biosynthesis were found between the young fruit of the two cultivars, indicating that the mutation-induced change in expression could occur far earlier than the emergence of the phenotype. Anthocyanins are end products of the flavonoid biosynthetic pathway; our finding of upregulation of almost all of this pathway's genes, from the upstream *CHS* to the end gene *UFGT*, during “Purple Peel” fruit ripening suggests that fundamental transcriptional regulation of the flavonoid and pigment biosynthetic pathways could be a major factor in the mutation, coordinating gene expression, fruit coloration, and the accumulation of flavonoid intermediates and procyanidins. In crabapple cultivars with dark red, pink and white petal colors, *CHS* has been found responsible for the red coloration (Tai et al., 2014). Upstream pathway expression regulation has also been reported in arctic mustard flowers, which have a broad range of purple to white petal color polymorphisms; in the white-flowered individuals, *CHS* was significantly repressed, whereas the expression of other structural genes in the anthocyanin biosynthetic pathway was similar to that in the colored individuals (Dick et al., 2011). The enzymes DFR and LAR are shared by the anthocyanin and flavanone biosynthetic pathways. DFR from different plants has specific substrate biases for dihydroquercetin, dihydrokaempferol and dihydromyricetin (Hua et al., 2013; Saito et al., 2013). LAR belongs to the reductase–epimerase–dehydrogenase family and the short-chain dehydrogenase/reductase superfamily, and each of the LARs has a specific C-terminal domain which may have different substrate specificity (Tanner et al., 2003). From our metabolome and transcriptome data, it seems that fig DFRs and LARs favor dihydroquercetin to produce leucocyanidin and catechin, rather than afzelechin and gallocatechin synthesis (Figure 3); thus, only cyanidin glycosides were the dominant anthocyanins, as with the B-type procyanidin in fig fruit (Table 1).

### Transcription factors in fruit color formation and ripening

Our finding of upregulation of most or all of the biosynthesis genes in the mutant fruit suggests mutation of a transcription factor. MYBs play a critical role as key transcription factors for all of the anthocyanin biosynthetic pathway genes or for the regulation of single key genes in fruit and flower color formation (Kobayashi et al., 2004; Espley et al., 2007; Tai et al., 2014). In apple, *CHS* is positively regulated by *MYB4* and *MYB5* expression (Clark and Verwoerd, 2011), whereas strawberry *FcMYB1* switches anthocyanins and flavonoid-derived compound accumulation on and off (Salvatierra et al., 2013). Loss of the MYB cis-element in the *CHS* promotor leads to white crabapple morphs (Dick et al., 2011). In our study, differentially expressed *MYB*s were recruited in the “Purple Peel” fig (Table 3, Figure 4), indicating that MYBs in the MBW complex are key regulators of the pathway of anthocyanin and flavonoid biosynthesis in fig.

### Hypothesized nature of the fig purple mutation

Red and black dominate the color spectrum of bird-dispersed fruit worldwide (Willson and Whelan, 1990). Anthocyanin synthesis and pigmentation can be regarded as the wild type for the fruit color trait. In grapes, white cultivars are thought to be mutations of red cultivars (Boss et al., 1996; Kobayashi et al., 2004; Hichri et al., 2011b), and all of the green grape cultivars have a common origin (Walker et al., 2007). The small seeds contained inside the fig syconia are dispersed by birds. We therefore assume that figs with a dark peel are the wild type, those with a green peel represent a color mutation, and the “Purple Peel” mutant of “Green Peel” can be regarded as a reverse mutation, regaining the wild-type trait.

Understanding the nature of the green-color fruit as a mutant of the wild type could facilitate analysis of the mechanism underlying the reverse mutation. Any functional loss of key enzymes in the anthocyanin biosynthetic pathway could lead to a green or white mutation, such as via insertion in the structural genes, and turned off or repressed structural gene expression by MYB transcription factors associated with the components of the MBW complex (Feller et al., 2011; Petroni and Tonelli, 2011; Tai et al., 2014). A large number of publications have demonstrated MYB family transcription factors as key regulators in phenylpropanoid, flavonoid, anthocyanin and proanthocyanidin biosynthesis (Falcone Ferreyra et al., 2012; Verdier et al., 2012; Liu et al., 2015; Xu et al., 2015). Moreover, studies with different fruit have revealed conserved components of the regulatory complex controlling anthocyanin biosynthesis in all higher plants, including conserved cis-regulation elements in promotors of key genes of the pathways (Quattrocchio et al., 1993; Koch et al., 2001; Stracke et al., 2007; Dick et al., 2011). The function and expression level of MYBs could be significantly affected by different types of mutations. A single amino-acid substitution in the R2 domain of *VvMYB5b* was found to affect the protein's ability to activate the transcription of flavonoid genes (Hichri et al., 2011b). A retrotransposon insertion in grape *mybA1* blocks the gene's expression, leading to loss of pigmentation in white grape cultivars (Kobayashi et al., 2004). In our study, differential expression of both transposons and retrotransposons was recorded, and a significant upregulation trend in a large number of reverse transcriptase, integrase and gag sequences was revealed in the “Green Peel” as compared to its purple mutant (Supplementary Table 4), suggesting that “Green Peel” is a retrotransposon insertion mutation.

In grapes, *VvMYBA1* and *VvMYBA2* have different on/off switch mechanisms: Gret1 retrotransposon insertion in the promoter of *VvMybA1* switches off *VvMybA1* expression, whereas a non-synonymous single-nucleotide polymorphism present in the coding region switches off the function of *VvMybA2* and leads to white grape berries (Kobayashi et al., 2004; Walker et al., 2007). In our case, *MYB*s, together with the changes in transposon and retrotransposon activation, could be candidates for the “Purple Peel” fig mutation from its “Green Peel” progenitor (Ramsay et al., 2003).

In summary, this combined metabolome and transcriptome study gives us a picture of modulated anthocyanin and flavanoid expression in the “Purple Peel” fig mutant, revealing the large-scale changes in nutritionally important compounds and gene expression in a horticultural mutation with a single phenotypic attribute. Our results provide new information on the anthocyanidin, flavonol and procyanidin metabolites of fig and the global transcriptional changes in fig color regulation, secondary metabolism pathways and regulators in fruit ripening and quality formation.

## Ethics statement

The study was approved by fig cooperatives in Weihai City, Shandong Province in China.

## Author contributions

HM and SC designed the experiments. ZW and YC conducted the experiments and analyzed the results. ZW, YC, AV, SC, and HM prepared the manuscript. All authors have read and approved the manuscript for publication.

### Conflict of interest statement

The authors declare that the research was conducted in the absence of any commercial or financial relationships that could be construed as a potential conflict of interest.

## Abbreviations

4CL: 4-Coumarate:coenzyme A ligase
ANS: Anthocyanidin synthase
bHLH: Basic helix-loop-helix
CHI: Chalcone isomerase
CHS: Chalcone synthase
COG: Clusters of orthologous groups of proteins database
DEG: Differentially expressed gene
DFR: Dihydroflavonol 4-reductase
F3H: Flavanone 3-hydroxylase
F3′H: Flavanone 3′-hydroxylase
LAR: Leucoanthocyanidin reductase
PAL: Phenylalanine ammonia-lyase
UFGT: UDP-glucose: flavonoid 3-O-glucosyltransferase.
