# Supplementary material for: Regulation of Fig (Ficus carica L.) Fruit Color: Metabolomic and Transcriptomic Analyses of the Flavonoid Biosynthetic Pathway
Source: Front Plant Sci. 2017 Nov 20;8:1990. doi: 10.3389/fpls.2017.01990 (PMC5701927; doi:10.3389/fpls.2017.01990)

Regulation of fig (*Ficus carica* L.) fruit color: metabolomic and transcriptomic analyses of the flavonoid biosynthetic pathway

Ziran Wang[1], Yuanyuan Cui[1], Alexander Vainstein[2], Shangwu Chen[3], Huiqin Ma[1,*]

[1] Department of Fruit Tree Sciences, College of Horticulture, China Agricultural University, Beijing 100193, China

[2] Institute of Plant Sciences and Genetics in Agriculture, The Robert H. Smith Faculty of Agriculture, Food and Environment, The Hebrew University of Jerusalem, Rehovot 76100, Israel

[3] College of Food Science and Nutrition Engineering, China Agricultural University, Beijing 100083, China

*Correspondence:
Huiqin Ma
hqma@cau.edu.cn

Supplementary Table 1 Primer sequences of genes used for quantitative RT-PCR verification

Gene Name	**Seq ID**	**Forward Primer (5’→3’)**	**Reverse Primer (5’→3’)**
Actin	Reference gene	GCCATTCAAGCCGTGCTTT	TGGGAACAGTGTGGCTGACA
FcMYB-1	c31006_g1	ACGGAAATGGCTGACTTGAG	TCTGCTGTTCTCCCTGGAAT
FcMYB-2	c41017_g1	CTTCGTCCTCCTCGTCGTAG	GATCGGTTATCCCGACTTCA
FcMYB-3	c47009_g3	GCAATTGCATTCAAGGGTTT	GCCTTCCAGACACCAAATGT
FcbHLH	c5179_g1	TACCACCACCACTCCTCCTC	CCTCCTTGCCCTAACATGAA
FcPAL	c34449_g3	GCAAGCCTTGAACTCTCCAC	GGTTCTGCGAGAAGGATCTG
FcC4H-1	c39884_g1	AGTCATGGACGCGTAAAACC	CTGGAATCATCATCGCATTG
FcC4H-2	c39884_g2	CGGAATGTTCCTGACTTCGT	CGTCGTCTTCGACATCTTCA
Fc4CL	c41047_g2	GCCCAGATCATGAAAGGGTA	TACTGCCGCATCTGCTATTG
FcCHS	c46769_g2	CCGTGAAGTTGGGCTTACAT	AAACCACACTTGGCTTCCAC
FcCHI-1	c658_g1	GTAACGGGTCCGTTTGAGAA	GTTAAAGAACCGGACGGTGA
FcCHI-2	c47235_g1	CACTTCCCTTCTTCCCCTTC	TGGTTGATGAAATCCCCTTC
FcF3H	c43823_g1	GTCGGTCGGGTATTCTTTGA	TAGATGGAGGAGCCATGAGG
FcF3’H-1	c42263_g3	GATCCGCCACCCTAAAATCT	GGATGTGGTAGCCGTTGACT
FcF3’H-2	c32643_g1	GTGGCGGAGCAGTTCCTTA	GAGGTGAACGGTGGTGATCT
FcFLS	c43823_g1	GGCGTCACCGTACCTGTAAT	AGAATACCCGACCGACCTCT
FcDFR	c18574_g2	AGGTGGAGGTAAGAGCAGCA	GATAGCCCATGCCACAACTT
FcLAR	c31753_g1	CGATGCCAGCAACAAAGTAA	GAGGAATCTGGTGTGCCCTA
FcANR	c43323_g1	CTTCGTCCACAACCAAACCT	CTTTGCCTCAGAGGATCCAG
FcANS	c59676_g1	CATCATGCACATTGGTGACA	GTGCTGGATATGCTGCTGAA
FcUFGT-1	c78174_g2	CGGAGAACACGGAGAAGAAG	ATTCCCCTCCAAATTCCAAC

FcUFGT-2	c66027_g1	CAGTGTCGTTTGCTGCAGAT	AAGGAAGTCAACGGCGAGTA

Supplementary Table 2 Summary of the sequencing and *de novo* assembly

Sequences	GY	PY	GM	PM
**Before trimming**				
Total nucleotides (bp)	4,866,846,986	3,884,679,020	5,001,699,397	4,203,482,991
Number of raw reads	32,342,640	25,821,256	33,232,728	27,935,164
Q20 percentage (%)	96.78	96.41	96.82	96.43
**After trimming**				
Total nucleotides (bp)	4,557,694,436.5	3,634,066,244.5	4,693,036,320.5	3,940,040,351
Number of raw reads	31,591,009	25,146,641	32,429,280	27,147,120
Q20 percentage (%)	98.37	98.25	98.34	98.19
**After assembly**				
Number of transcripts of combined data	96,158			
Number of unigenes of combined data	79,355			
Total nucleotides of transcripts (bp)	76,582,246			
Total nucleotides of unigenes (bp)	54,205,110			
Mean length of transcripts (bp)	796.42			
Mean length of unigenes (bp)	683.07			
GC content of transcripts (%)	41.79			
GC content of unigenes (%)	41.71			
N50 of unigenes (bp)	1236			
N90 of unigenes (bp)	261			

GY, 'Green Peel' young fruit; PY, 'Purple Peel' young fruit; GM, 'Green Peel' mature fruit; PM, 'Purple Peel' mature fruit.

Supplementary Table 3 Expression profiles of the major differentially expressed transcription factor (TF) genes in young and mature stages of 'Purple Peel' and 'Green Peel' fig

a.

**Seq_id**	**LogFC(PY/GY)**	***P*-value**	**GY (mean FPKM)**	**PY (mean FPKM)**	**TF family**	**Regulate**
c29668_g2	5.25	2.95E-114	97.29	3699.93	AP2/ERF	up
c44787_g1	3.76	4.34E-07	0.09	2.5	AP2/ERF	up
c46866_g2	2.73	3.82E-16	19.58	130.48	AP2/ERF	up
c59585_g1	2.14	8.18E-21	384.36	1692.83	AP2/ERF	up
c41479_g1	2.12	5.59E-23	59.47	259.14	AP2/ERF	up
c41779_g1	-2.04	5.69E-19	70.23	16.94	AP2/ERF	down
c36532_g1	-2.29	5.69E-03	3.92	0.72	AP2/ERF	down
c31043_g1	-2.36	4.21E-03	9.82	1.83	AP2/ERF	down
c35689_g1	-2.36	1.89E-04	3.46	0.59	AP2/ERF	down
c59932_g1	-2.43	9.03E-17	41.13	7.53	AP2/ERF	down
c42243_g1	-3.8	2.63E-35	130.69	9.3	AP2/ERF	down
c27822_g1	-3.86	5.98E-28	29.81	1.95	AP2/ERF	down
c36532_g2	-4.43	4.63E-11	8.27	0.29	AP2/ERF	down
c35625_g1	-4.84	2.42E-10	9.06	0.22	AP2/ERF	down
c17499_g1	-6.55	8.35E-14	9.25	0	AP2/ERF	down
c37406_g1	3.91	5.96E-46	38.84	585.93	DIVARICATA	up
c72503_g1	2.4	2.05E-28	30.11	159.87	DIVARICATA	up
c44885_g4	2.24	2.67E-15	7.12	34.08	DIVARICATA	up
c47573_g1	-2.17	9.31E-03	13.93	3.01	DIVARICATA	down
c26517_g2	2.26	8.39E-03	2.65	13.05	HSF	up

c43194_g3	2.41	3.68E-11	1.85	10.28	HSF	up
c13473_g1	2.26	6.94E-03	0.44	2.48	MADS-box	up
c2721_g1	2.83	1.48E-33	29.45	210.5	WRKY	up
c41515_g1	2.18	5.34E-09	4.98	22.98	WRKY	up
c42471_g1	-3.03	6.00E-30	41.02	4.94	WRKY	down
c47602_g1	-3.06	3.49E-42	47.51	5.62	WRKY	down

b.

**Seq_id**	**LogFC(PM/GM)**	***P*-value**	**GM (mean FPKM)**	**PM (mean FPKM)**	**TF family**	**Regulate**
c46520_g1	2.36	1.82E-16	7.79	40.41	AP2/ERF	up
c44787_g1	2.4	1.69E-03	0.25	1.72	AP2/ERF	up
c29668_g2	2.82	1.24E-25	299.82	2116.89	AP2/ERF	up
c42243_g1	3.16	9.51E-28	8.8	79.34	AP2/ERF	up
c34225_g1	2.25	3.02E-05	5.59	26.91	AP2/ERF	up
c46866_g2	3.38	7.23E-07	0.51	6.19	AP2/ERF	up
c15752_g1	3.1	3.84E-04	1.85	16.68	AP2/ERF	up
c20775_g2	2.83	1.12E-04	0.82	6.43	AP2/ERF	up
c31910_g1	2.25	1.89E-18	33.14	157.71	AP2/ERF	up
c45384_g1	2.2	4.22E-12	7.3	33.91	HSF	up
c26517_g2	3.05	2.30E-04	1.69	14.73	HSF	up
c43194_g3	3.75	3.96E-41	6.35	86.71	HSF	up
c11592_g1	-2.21	6.46E-04	3.63	0.71	WRKY	down
c391_g1	-2.91	7.72E-04	2.12	0.2	WRKY	down

GY, 'Green Peel' young fruit; PY, 'Purple Peel' young fruit; GM, 'Green Peel' mature fruit; PM, 'Purple Peel' mature fruit.
FDR $\leq$ 0.001 and absolute value of log2 ratio $\geq$ 2 (2-fold) were used as the threshold for differential expression.

Supplementary Table 4 Expression profiles of differentially expressed transposon-related genes in young and mature stages of 'Purple Peel' and 'Green Peel' fig fruit

Seq_id	LogFC (PY/GY)	*P*-value	GY (mean_FPKM)	PY (mean_FPKM)	Annotation
**Transposon**					
c47009_g3	6.49	5.13E-154	3.13	289.27	DDE_Tnp_4, Myb_DNA-bind_3
c40445_g3	-1.6	4.86E-03	2.31	0.7	DDE_Tnp_4, Myb_DNA-bind_3
c44621_g1	6.46	2.60E-03	0.06	14.27	DEDD_Tnp_
c42314_g1	-2.07	1.17E-26	146.39	34.74	DDE_Tnp_4
c43959_g1	-1.55	2.28E-03	2.59	0.82	DDE_Tnp_4|
c30056_g1	-1.09	5.19E-03	2.99	1.35	MuDR family transposase
c33254_g1	1.23	6.00E-04	6.65	15.75	MuDRA-like transposase
	Total fold, 7.87				
**Reverse transposon**					
c47285_g1	6.81	6.17E-05	0.37	53.25	Reverse transcriptase
c36014_g1	4.29	5.56E-05	0	1.86	Reverse transcriptase
c44832_g2	4.18	2.44E-10	0.26	6.51	Reverse transcriptase-like
c8785_g1	3.28	3.63E-07	0.21	2.92	Reverse transcriptase
c41811_g1	2.03	7.85E-17	26.6	108.62	Reverse transcriptase-like
c66217_g1	-3.45	1.81E-04	1	0	Reverse transcriptase, Putative gag-pol polyprotein
c61362_g1	-2.91	1.43E-02	1.19	0.07	Reverse transcriptase-like
c31218_g1	-2.74	8.31E-08	1.87	0.2	Reverse transcriptase
c23719_g1	-2.69	1.75E-02	0.95	0.06	Reverse transcriptase, Gag-int-pol protein
c30372_g1	-2.13	7.63E-03	1.89	0.36	Reverse transcriptase-like

c40198_g1	-2.06	1.29E-09	3.75	0.83	Reverse transcriptase
c47142_g1	-2.06	1.57E-02	0.94	0.15	Reverse transcriptase
c46876_g1	-1.79	9.45E-04	4.71	1.29	Reverse transcriptase
c44735_g1	-1.71	1.20E-20	99.39	30.3	Reverse transcriptase
c47013_g1	2.38	3.85E-22	4	21.16	Integrase core domain
c47203_g3	1.16	1.75E-05	14.69	32.93	Integrase core
c47203_g5	-2.14	1.04E-02	4.87	1.03	Integrase core domain
c59762_g1	-3.46	1.52E-05	1	0	Retrotran_gag_3
c46291_g1	-1.89	5.84E-04	2.07	0.49	gag_pre-integrs
c45677_g1	-1.87	6.87E-04	3.05	0.76	gag-pre-integrase domain
c47270_g2	-1.61	8.09E-03	1.05	0.28	gag_pre-integrs
c47275_g1	-1.4	1.87E-02	2.36	0.83	Retrotransposon gag protein
	Total fold, -9.78				

Mature fruit

**Seq_id**	**LogFC (PM/GM)**	***P*-value**	**GM (mean_FPKM)**	**PM (mean_FPKM)**	**Annotation**
**Transposon**					
c47009_g3	2.25	3.04E-17	72.97	348.54	DDE_Tnp_4, Myb_DNA-bind_3
c42314_g1	2.03	6.20E-16	42.03	171.81	DDE_Tnp_4
c38877_g1	-1.32	3.41E-04	11.62	4.59	DDE_Tnp_4
c78571_g1	-1.09	3.94E-04	17.19	8	DDE_Tnp_4
c13237_g1	-1.23	1.09E-04	9.46	3.99	Dimer_Tnp_hAT
c31666_g1	5.61	1.25E-53	0.38	23.3	MuDR family transposase
c30398_g1	-1.11	1.78E-03	7.34	3.34	MuDR family transposase

	Total fold, 5.14				
**Reverse transposon**					
c44832_g2	2.45	1.45E-03	0.25	1.79	Reverse transcriptase-like
c41811_g1	1.2	5.12E-06	59.41	136.59	Reverse transcriptase-like
c33584_g1	-1.96	6.43E-03	1.45	0.3	Reverse transcriptase-like
c36014_g1	2.24	3.12E-03	0.45	2.47	Reverse transcriptase
c40198_g1	-1.99	2.86E-07	4.44	1.04	Reverse transcriptase
c25773_g3	-3.11	1.57E-03	1.67	0.1	Reverse transcriptase
c45494_g1	-2.81	2.17E-03	1	0.06	Reverse transcriptase
c46549_g1	-2.04	4.40E-03	1.76	0.35	Reverse transcriptase
c45804_g2	-1.9	1.17E-03	3.28	0.81	Reverse transcriptase
c47142_g1	-1.84	4.46E-03	3.67	0.95	Reverse transcriptase
c45248_g1	-1.8	5.69E-03	0.87	0.18	Reverse transcriptase
c79265_g1	-1.78	1.44E-03	1.29	0.3	Reverse transcriptase
c46976_g4	-1.77	7.11E-03	1.76	0.45	Reverse transcriptase
c37543_g2	-1.75	5.78E-03	1.49	0.37	Reverse transcriptase
c36794_g1	-1.69	3.37E-03	1.65	0.44	Reverse transcriptase
c43753_g1	-1.41	6.12E-04	7.63	2.82	Reverse transcriptase
c47174_g1	-1.72	7.01E-04	3.23	0.91	Reverse transcriptase, gag_pre-integrs
c45112_g3	-1.52	3.77E-03	2.31	0.74	Reverse transcriptase, gag-integrase domain
c47013_g1	1.59	4.34E-09	43.57	131.4	Integrase core domain

c44625_g1	-2.18	1.58E-07	2.76	0.53	integrase core domain|
c47066_g1	-1.76	6.69E-04	2.23	0.59	Integrase core domain
c47203_g5	-1.7	3.02E-05	15.36	4.67	Integrase core domain
c47275_g1	-2.85	9.07E-08	5.88	0.73	Retrotransposon gag protein
c59762_g1	-2.59	4.57E-16	59.38	9.75	Retrotran_gag_3
c11238_g1	-1.6	1.34E-06	12.47	4.06	Retrotransposon gag protein
c47270_g2	-2.66	1.81E-11	5.58	0.8	gag_pre-integrs
c59762_g1	-2.59	4.57E-16	59.38	9.75	Retrotran_gag_3
c46291_g2	-2.02	1.33E-04	3.09	0.69	gag_pre-integrs
c41066_g1	-2.18	1.49E-03	1.87	0.33	gag-integrase
c45234_g5	-1.73	3.54E-05	4.92	1.41	Retrotran_gag_2
c45809_g1	-1.67	1.76E-06	10.06	3.1	gag-integrase domain
c46291_g1	-1.58	4.74E-04	4.31	1.37	gag-integrase domain
c46800_g3	-1.53	2.41E-04	10.92	3.73	Retrotran_gag_2
c45677_g1	-1.53	1.59E-03	4.11	1.36	gag-integrase domain
c42555_g3	-1.4	3.15E-05	11.88	4.45	Retrotran_gag_2
	Total fold, -52.0				

GY, 'Green Peel' young fruit; PY, 'Purple Peel' young fruit; GM, 'Green Peel' mature fruit; PM, 'Purple Peel' mature fruit.
FDR ≤ 0.001 and absolute value of log2 ratio ≥ 2 (2-fold) were used as the threshold for differential expression.

Supplementary Figure legends:

Supplementary Figure 1 Representative MRM profiles of fig fruit peel samples. GY, ‘Green Peel’ young fruit; GM, ‘Green Peel’ mature fruit; PY, ‘Purple Peel’ young fruit; PM, ‘Purple Peel’ mature fruit.

Supplementary Figure 2 Expression patterns of secondary metabolites revealed by metabolomic analysis. GY, ‘Green Peel’ young fruit; GM, ‘Green Peel’ mature fruit; PY, ‘Purple Peel’ young fruit; PM, ‘Purple Peel’ mature fruit; a, b and c represent the three biological replicates.

Supplementary Figure 3 GO classification of differentially expressed genes of young and mature stages of 'Purple Peel' and 'Green Peel' fig. GY, ‘Green Peel’ young fruit; PY, ‘Purple Peel’ young fruit; GM, ‘Green Peel’ mature fruit; PM, ‘Purple Peel’ mature fruit.

Supplementary Figure 4 COG functional classification of differentially expressed genes of young and mature stages of 'Purple Peel' and 'Green Peel' fig. GY, ‘Green Peel’ young fruit; PY, ‘Purple Peel’ young fruit; GM, ‘Green Peel’ mature fruit; PM, ‘Purple Peel’ mature fruit.

Supplementary Figure 5 Differentially expressed bHLH genes in the young and mature stages of 'Purple Peel' and 'Green Peel' fig. A. Comparison between young stages. B. Comparison between mature stages. GY, ‘Green Peel’ young fruit; PY, ‘Purple Peel’ young fruit; GM, ‘Green Peel’ mature fruit; PM, ‘Purple Peel’ mature fruit.

Supplementary Figure 6 Correlation of fold changes of 20 differentially expressed genes by RNA-Seq and qRT-PCR analysis. GY, ‘Green Peel’ young fruit; PY, ‘Purple Peel’ young fruit; GM, ‘Green Peel’ mature fruit; PM, ‘Purple Peel’ mature fruit.

Supplementary Figure 1

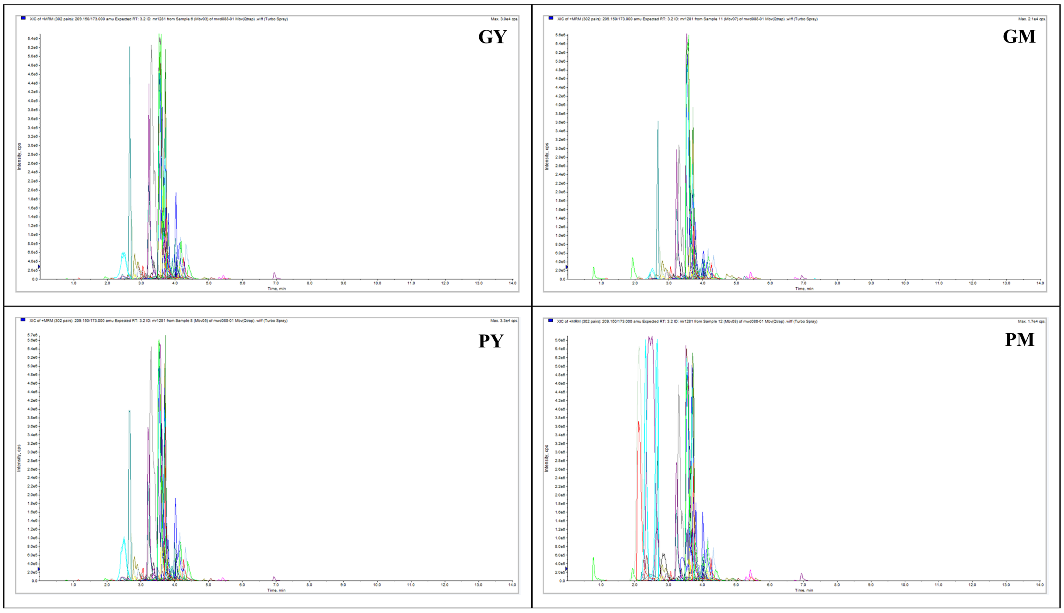

Supplementary Figure 2

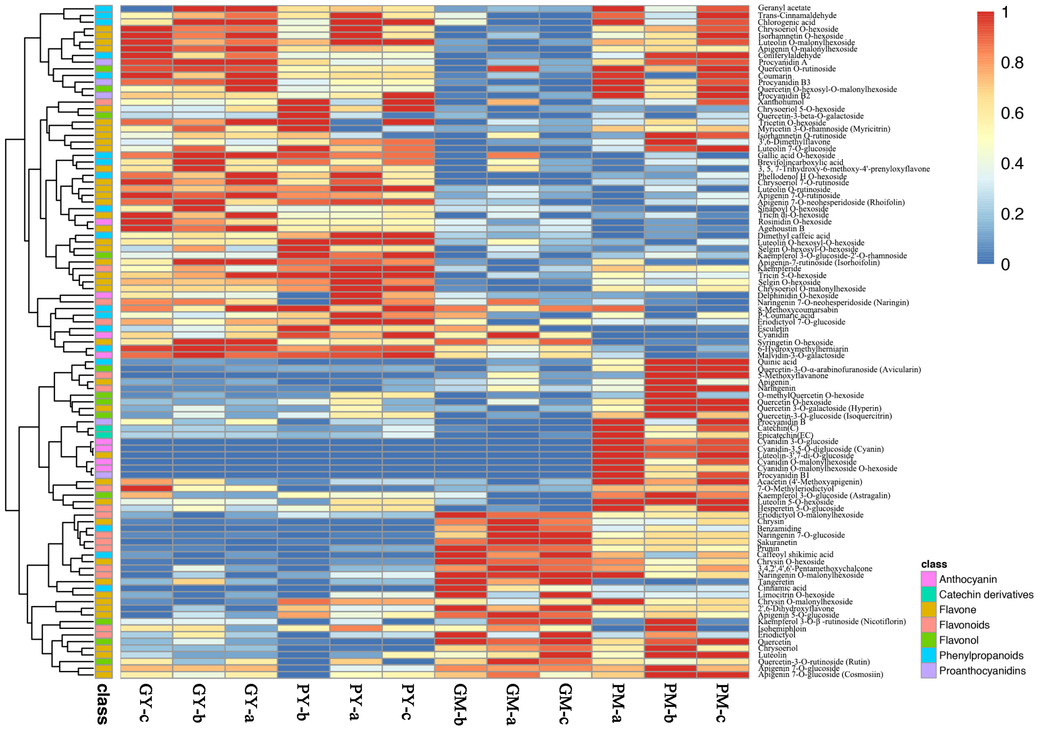

Supplementary Figure 3

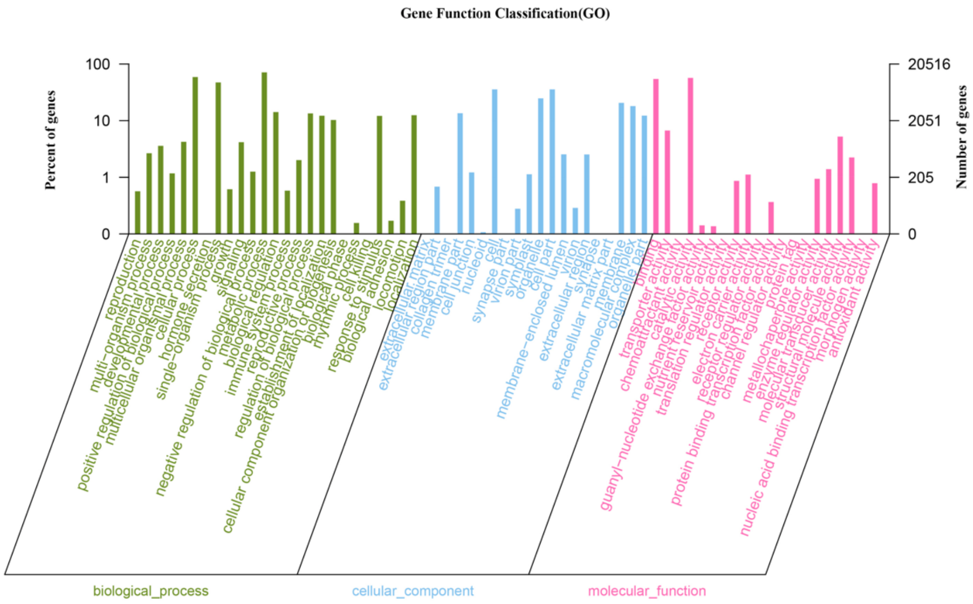

Supplementary Figure 4

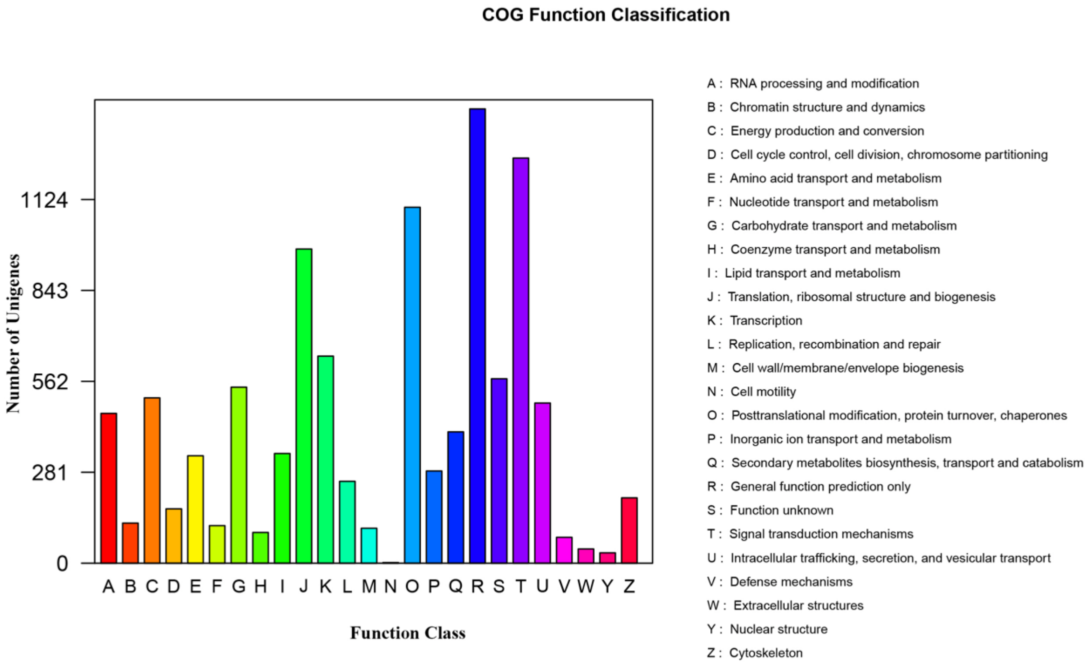

Supplementary Figure 5

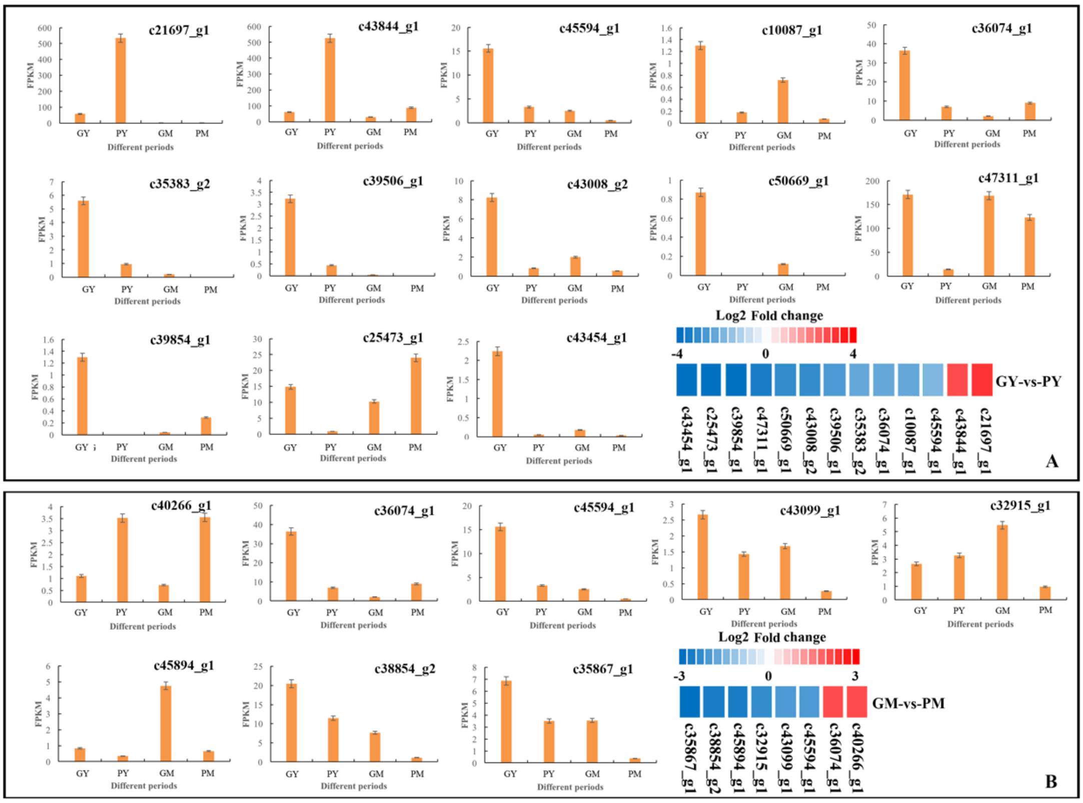

Supplementary Figure 6

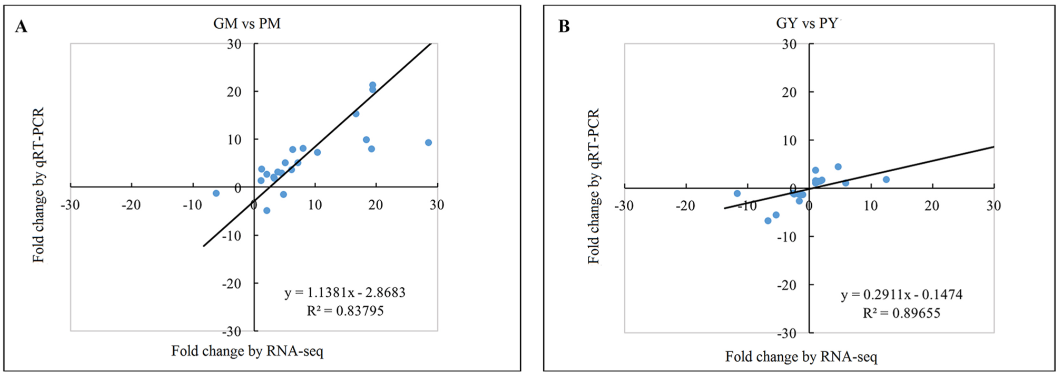

Supplement: Supplementary file 1 [file Presentation1.PDF]
